# When Disability Begins Matters: Later-Onset Disability and Masticatory Discomfort in Older Adults

**DOI:** 10.1093/geroni/igaf122.3684

**Published:** 2025-12-31

**Authors:** Chan-Young Park, Nam-Hee Kim

**Affiliations:** Yonsei University, Yonsei University, Kangwon-do, Republic of Korea; Yonsei University, Wonju, Kangwon-do, Republic of Korea

## Abstract

This study investigates the effect of disability onset timing on masticatory discomfort among older adults, drawing on nationally representative data from the 2023 Korean National Disability Survey. We classified 6,180 adults aged 50 and older into two groups: Aging with Disability (AWD; disability onset before age 50) and Disability with Aging (DWA; onset at age 50 or later). Masticatory discomfort was measured via self-report, and weighted logistic regression was used to identify predictors, adjusting for age, gender, disability type and severity, oral health behaviors, and healthcare access. Masticatory discomfort was significantly more prevalent in the DWA group (37.1%) compared to the AWD group (22.2%, p < 0.001). Even after full adjustment, DWA status remained an independent predictor (odds ratio [OR] 1.23; 95% confidence interval [CI] 1.04–1.45). Other significant predictors included infrequent tooth brushing (once per day or less; OR 1.84, p < 0.001), no dental checkups in the past year (OR 1.46, p < 0.001), unmet healthcare needs (OR 1.73, p < 0.001), older age (age 80 or above; OR 3.23, p < 0.001), lower income (OR 2.06, p < 0.001), and lower educational attainment (elementary or less; OR 1.39, p = 0.002). These findings suggest that later-life disability onset increases vulnerability to oral health problems, likely due to limited adaptation time, weaker coping strategies, and barriers to care. The results highlight the need for life-course–sensitive oral health interventions and suggest that the timing of disability onset should be a key consideration in geriatric care and public health policy.

